# Magnetic Heating of Nanoparticles Applied in the Synthesis of a Magnetically Recyclable Hydrogenation Nanocatalyst

**DOI:** 10.3390/nano10061142

**Published:** 2020-06-10

**Authors:** Sašo Gyergyek, Darja Lisjak, Miloš Beković, Miha Grilc, Blaž Likozar, Marijan Nečemer, Darko Makovec

**Affiliations:** 1 Department for Materials Synthesis, Jožef Stefan Institute, Jamova 60, 1000 Ljubljana, Slovenia; darja.lisjak@ijs.si (D.L.); darko.makovec@ijs.si (D.M.); 2 Institute of Electrical Power Engineering, Faculty of Electrical Engineering and Computer Science, University of Maribor, Koroška 46, 2000 Maribor, Slovenia; milos.bekovic@um.si; 3 Department of Catalysis and Chemical Engineering, National Institute of Chemistry, Hajdrihova 19, 1000 Ljubljana, Slovenia; miha.grilc@ki.si (M.G.); blaz.likozar@ki.si (B.L.); 4 Department for Low and Medium Energy Physics, Jožef Stefan Institute, Jamova 60, 1000 Ljubljana, Slovenia; marijan.necemer@ijs.si

**Keywords:** catalyst, ruthenium, biomass, induction heating, magnetic separation, magnetic heating

## Abstract

Utilization of magnetic nanoparticle-mediated conversion of electromagnetic energy into heat is gaining attention in catalysis as a source of heat needed for a substrate’s chemical reaction (electrification of chemical conversions). We demonstrate that rapid and selective heating of magnetic nanoparticles opens a way to the rapid synthesis of a nanocatalyst. Magnetic heating caused rapid reduction of Ru^3+^ cations in the vicinity of the support material and enabled preparation of a Ru nanoparticle-bearing nanocatalyst. Comparative synthesis conducted under conventional heating revealed significantly faster Ru^3+^ reduction under magnetic heating. The faster kinetic was ascribed to the higher surface temperature of the support material caused by rapid magnetic heating. The nanocatalyst was rigorously tested in the hydrotreatment of furfural. The activity, selectivity and stability for furfural hydrogenation to furfuryl alcohol, a valuable biobased monomer, remained high even after four magnetic recycles.

## 1. Introduction

Renewable electricity will play an important role in the near future decarburization of human society. Due to variable availability, new strategies for its use must be developed in addition to storage in batteries. The direct conversion of surplus renewable electricity into chemicals such as fuels, monomers, solvents etc., a concept that the German Chemical Engineering Association named power-to-chemicals (P2C), is becoming an important additional strategy [1]. The current focus is on electrocatalytic processes [1]; however, it makes sense to develop other electricity-based technologies. Magnetic nanoparticles (MNPs) heat rapidly and extensively under the influence of an alternating magnetic field, a phenomenon extensively studied for cancer treatment [2,3]. The application of this principle to drive chemical reactions has only recently been demonstrated [4,5,6,7,8,9,10]. Kirschning et al. showed for the first time that magnetic nanoparticles in the Alternating Current (AC) field of low-to-medium frequency can heat sufficiently to drive technologically important chemical reactions in continuous operation and significantly improve yields compared to conventional heating [4]. Chaudret et al. developed complex MNP-bearing catalytic Ru nanoparticles with superior magnetic properties, resulting in an exceptionally high specific absorption rate (SAR), the figure-of-merit indicating the amount of heat the material generates in the AC field of the specified frequency and amplitude [7,8]. Complex nano-objects have been used to demonstrate magnetically induced batch CO and continuous CO_2_ hydrogenation [7,8]. It has been pointed out recently that the ability to use an AC field to deliver heat where needed is an enabling technology suited to push many catalytic reactions beyond the reactor heat transfer limits, to the limits of process kinetics [9]. Overall, the claimed advantages of the technology are manifold: more favorable energy balance, process intensification, reactor setup simplification, reduced safety issues, minor operational costs and increased process productivity [9]. In addition to novel applications in catalysis, magnetic induction heating has been proven in the synthesis of metal–organic framework MNP composites (MOF-MNPs) [11] and in its application in magnetic induction swing adsorption (MISA) [12]. Magnetically induced selective heating of MNPs dispersed in the MOF precursor solution has shown an advantage in the increased kinetics of formation of MOF over conventional heating [11]. Furthermore, the yield and size of composite MOFs can be controlled by reaction time, MNP concentration and strength of magnetic field [11]. Supported precious metal nanoparticles, such as ruthenium (Ru) nanoparticles, are often used in the catalytic conversion of biomass into value-added chemicals [13]. Carbon as a support material for catalysts has long been relevant due to its commercial availability, thermochemical stability, low density and simple preparation from various natural sources, such as biomass, sugars and fatty acids [14,15]. Catalytic nanoparticles are typically deposited on a support material using methods that fall broadly into two categories: impregnation of support [16] and precipitation in suspension of the support material [16,17]. The methods of the first category are based on adsorption of metal cations on the surface of the support from the solution, followed by drying and reduction, or on adsorption of previously synthesized metal nanoparticles. Methods from the second category are based on the reduction of the metal precursor in the suspension of the support, causing the precipitation of metal nanoparticles and their deposition on the support’s surface. Recently, our group has developed a method for preparation of magnetically separable Ru-based nanocatalysts that shows remarkable activity for the hydrogenation/hydrodeoxygenation of the lignin monomer model compound eugenol [17,18]. The method is based on solvothermal reduction of Ru(acac)_3_ in the suspension of the magnetic carbon support by the environmentally benign solvent isopropanol.

Here, we applied for the first time the induction heating of magnetic nanoparticles incorporated within the graphitic material (support) to demonstrate the synthesis of a nanocatalyst. Specifically, induction heating of the magnetic nanoparticles caused the surface temperature of the support to rise rapidly, while the bulk of the liquid remained at a much lower temperature. A high enough temperature was achieved to cause a rapid reduction of Ru^3+^ and an exclusive deposition of Ru nanoparticles and atomic clusters on the surface of the support. The nanocatalyst showed a 100% conversion of furfural with 99% selectivity for valuable biobased monomer furfuryl alcohol, even after being recycled four times.

## 2. Materials and Methods

Iron (III) sulphate hydrate (Fe_2_(SO_4_)_3_ *x*H_2_O, (Sigma-Aldrich, St. Louis, MO, USA), iron (II) sulphate hydrate (FeSO_4_·7H_2_O 92%, Alfa-Aesar, Haverhill, MA, USA), ammonium hydroxide (NH_4_OH 25% solution, J.T. Baker, Radnor Township, PA, USA), citric acid monohydrate (ACS 99–102%, Alfa-Aesar), acetone (reagent grade, Carlo-Erba, Barcelona, Spain), glucose (d-glucose 99%, Alfa-Aesar), ruthenium (III) 2,4-pentadionate (Ru(C_5_H_7_O_2_)_3_ 97%, Sigma-Aldrich), 2-propanol (ACS reagent grade, VWR, Radnor, PA, USA), Ru/C (5 wt.% Ru, Sigma Aldrich) and furfural (99%, Sigma-Aldrich) were used as received.

The magnetic nanoparticle-containing support (MN-C) was prepared by modification of our previously reported route (see Supplementary Materials Section S1 for details) [17]. In short, the precursor particles were prepared by hydrothermal treatment of glucose dissolved in the aqueous suspension of citric-acid-coated magnetic iron oxide nanoparticles [17]. The precursor particles had a diameter of approximately 100–300 nm and contained homogeneously dispersed iron oxide nanoparticles (~10 nm in diameter) in the carbonaceous matrix [17]. The MN-C was prepared by annealing the precursor for 6 h at 600 °C in an Ar atmosphere. Magnetically mediated synthesis of the catalyst AC-Ru was conducted in a round bottom glass pressure vessel (see Supplementary Materials Section S1 for details, Table S1). The MN-C and 3 mL of 0.01 M Ru (III) 2,4-pentadionate were sealed, the vessel was placed in the centre of the inductor coil and the field of *μ*_0_*H* = 86 mT was turned on. After 19 min, the vessel was removed from the coil and in approximately 3 min cooled to room temperature when it was opened and the AC-Ru was separated using a handheld permanent magnet. The AC-Ru was washed 5 times with pure 2-propanol and dried in a vacuum at room temperature. The catalyst CH-Ru was synthesized under identical conditions, but using an oil bath heated to 150 °C as heat source. The synthesis proceeded for 30 min (see Supplementary Materials Section S1 for details). The hydrogenation of furfural was conducted in a pressure vessel (see Supplementary Materials Section S1 for details). Reaction conditions: 48 mg of catalyst, 0.1 g of furfural, 0.8 g of 2-propanol, H_2_ pressure 10 bar, 90 °C for 3 h. The AC-Ru catalyst was recycled 4 times (5 hydrogenations in total using the same catalyst) and the CH-Ru 1 time (2 hydrogenations in total using the same catalyst).

Scanning electron microscope (SEM) images of the AC-Ru were obtained using a Jeol JSM-7600F (Jeol, Tokyo, Japan) microscope. The sample was deposited on a conducting graphite tape and imaged without any further preparation. The X-ray powder diffraction pattern (XRD) was collected using a Siemens D5005 diffractometer (Siemens, Munich, Germany) with a monochromator in the diffracted beam. Quantitative phase analyses based on a Rietveld refinement of the XRD pattern of the MN-C were made using DIFFRACplus Topas® software (Siemens, Munich, Germany). A scanning transmission electron microscope (STEM) Cs-corrected Jeol ARM 200CF STEM (Jeol, Tokyo, Japan) operated at 80 kV was used to observe the AC-Ru and CH-Ru catalysts, deposited on a copper-grid-supported lacy carbon foil. During the analysis, High Angle Annular Dark Field (HAADF) and Bright Field (BF) detectors were used simultaneously at 68–180 and 10–16 mrad collection semi-angles, respectively. The Raman spectrum of the MN-C was recorded with a NT-MDT model Integra Spectra for Materials Science equipped with a confocal microscope (20× magnification) at room temperature. A 633-nm laser diode was used for the excitation. The scattered light was detected using a cooled Charge Coupled Device (CCD) camera and a 600 grooves/mm grating, and 10 spectra were accumulated, each acquired for 60 s to increase the signal-to-noise ratio. The spectra were analyzed offline using OriginPro 2015 64-bit software (OriginLabs, Northampton, MA, USA). Raman spectra were fitted with 5 Lorentzian functions. Nitrogen adsorption/desorption isotherm was measured for the sample at liquid-nitrogen temperature using a Nova 2000e (Quantachrome, Boynton Beach, FL, USA) nitrogen-sorption analyzer. Prior to the measurement, the sample was degassed over night at 120 °C in a vacuum. The surface area was calculated using the Brunauer–Emmett–Teller (BET) equation with the nitrogen-adsorption data in the *P*/*P_0_* range between 0.05 and 0.3 (7-point analysis). The room-temperature magnetization curve of the MN-C was measured with a vibrating-sample magnetometer (VSM) LakeShore 7307 VSM. Ru and Fe contents in MN-C, AC-Ru and CH-Ru were non-destructively determined using an energy dispersive X-ray fluorescence spectrometer (XRF) comprised of a Ge semiconductor detector (GLP-16195/10-P, ORTEC, Oak Ridge, TN, USA) with an energy resolution of 401 eV at 60 keV, a spectroscopy amplifier (M2020, Canberra, Meriden, CT, USA), an Analogue Digital Converter (M8075, Canberra) and a Personal Computer-based Multi Chanel Analyzer (S-100, Canberra). For the excitation, the annular Am-241 radioactive source (25 mCi, Isotope Products Laboratories, Santa Clara, CA, USA) was used. Quantification was performed utilizing the in-house developed QAES (quantitative analysis of environmental samples) software [19]. For the analysis, sample powders were diluted with cellulose and pressed into a pellet of 24 mm in diameter. The specific absorption rate (SAR) of the MN-C was determined from the measurements of temperature increase in the AC field. An amount of 0.25 mL of the suspension of 5 wt.% MN-C in isopropanol was placed in a plastic round-bottom 2-mL Eppendorf tube. The tube that was isolated from the surroundings by a polystyrene chamber was fitted in the center of the coil (Figures S1 and S2). The temperature at the middle of the sample was continuously measured every second using a fiber optical probe. The sample was exposed to the AC field for a time varying between 15 and 20 s. The heating curves were fitted with a linear function in the initial region where the increase in temperature was linear with time. SAR values were calculated using the expression:

(1) $$SAR=\frac{c_{p}}{w}{\left(\frac{dT}{dt}\right)}_{i}$$

where *c_p_* is the heat capacity of isopropanol (2.68 J/gK), *w* is the weight fraction of Fe in the MN-C in isopropanol and (d*T*/d*t*)_I_ is the slope of the linear fit in the initial region. The SAR can be interpreted as the amount of heat generated by the sample per gram of Fe incorporated within the MN-C was calculated, where the weight fraction of Fe in MN-C was determined by the XRF. Liquid products of the hydrogenations were analyzed by Gas Chromatography (GC-QMS) (Ultra 2010, Shimadzu, Kyoto, Japan) and ^1^H Nuclear magnetic resonance (NMR) (Spinsolve 60, Magritek, Wellington, New Zealand).

## 3. Results and Discussion 

**Figure 1:**
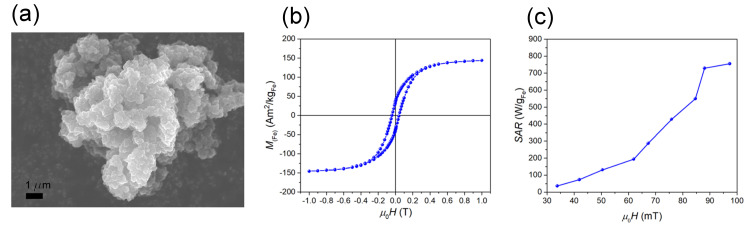
(**a**) SEM image of the room-temperature magnetization curve. (**b**) Specific absorption rate (SAR) (measured at 273 kHz) as a function of the AC-field amplitude (**c**) of the magnetic nanoparticle-containing support (MN-C).

The SEM analysis showed that the MN-C was in the form of agglomerates of micron-sized irregularly shaped particles (Figure 1a). The rough and bulgy surface of MN-C particles offered a large surface area of 258 m^2^/g and pore volume of 0.379 cm^3^/g. The partial decomposition of the organic part of the precursor during annealing led to a partial reduction of iron oxide nanoparticles to Fe^0^. A quantitative phase analysis based on the Rietveld refinement of the XRD pattern (Figure S3) showed that the MN-C consists of body centered cubic Fe nanoparticles (26.0 wt.%, with a crystallite size of 54 nm), spinel iron oxide nanoparticles (10.0 wt.%, 14 nm) and nanocrystalline graphite (64.0 wt.%, 5 nm). The total Fe content determined by XRF [19] was found to be 22.0 wt.%. The STEM analysis of the MN-C also revealed an irregular shape of the MN-C and a homogeneous distribution of the magnetic nanoparticles within the carbon matrix (Figure S4). Raman spectroscopy showed that the carbon matrix consists of nanocrystalline graphite, amorphous carbon phases and polyenes (Figure S5) [20,21,22,23,24,25]. The MN-C showed ferromagnetic behavior with a high saturation magnetization *M*_S_(Fe) = 148 Am^2^/kg_Fe_ (expressed per amount of Fe) (Figure 1b). The good magnetic properties, namely, high magnetization and relatively high magnetic susceptibility (Figure 1b), were reflected in the high SAR values (measured at 273 kHz) (Figure 1c). (For details, see Supplementary Materials Section S2, Figures S1, S2 and Table S2). The SAR increased with the AC-field amplitude non-linearly, showing an abrupt increase above approximately 65 mT and indicating a saturation above approximately 85 mT (Figure 1c). The highest SAR value of approximately 800 W/g_Fe_ was measured for the sample at an amplitude of 97 mT (Figure 1c).

**Figure 2:**
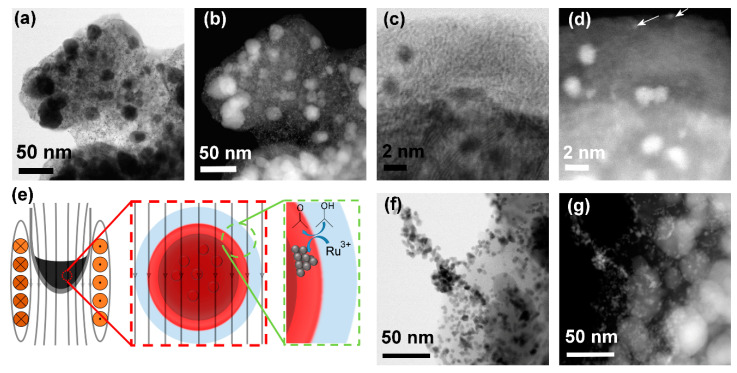
(**a**,**c**) Bright Field (BF) and (**b**,**d**) High Angle Annular Dark Field (HAADF) scanning transmission electron microscope (STEM) images of the nanocatalyst AC-Ru. (**e**) Schematic illustration of the magnetically mediated synthesis of the nanocatalyst AC-Ru. (**f**) BF and (**g**) HAADF STEM images of the nanocatalyst CH-Ru. Arrows in (**d**) show Ru cluster.

The MN-C was used to demonstrate the magnetically mediated synthesis of the nanocatalyst AC-Ru (Figure 2). The synthesis was conducted in a closed quartz pressure vessel, with the MN-C particles dispersed in a solution of Ru(C_5_H_7_O_2_)**_3_** in 2-propanol (see Supplementary Materials Section S2). The vessel was inserted into an inductor coil and exposed to the AC-magnetic field with an amplitude of *μ*_0_*H* = 86 mT at the frequency of 273 kHz. No stirring was applied, and the temperature of the liquid was monitored by measuring the wall temperature of the vessel with the IR camera. Within 19 min (Table S1), practically the entire Ru^3+^ was reduced and deposited on the surface of the support in an exceptionally homogeneous manner (Figure 2a,b). To check if the only source of heat was magnetic MN-C particles, the blank test was performed. The same amount of isopropanol was positioned at the same position within the coils as the MN-C sample. Applying the same AC-field amplitude (frequency of 273 kHz) for 30 min resulted in a minor, 3 °C increase of the temperature, indicating that the coils or any other part of the equipment do not contribute to the heating of the MN-C. Recently, it was shown that the metastable tetragonal Ru exhibits ferromagnetic behavior [26]. Comparing the SAR values (unpublished results) of similar materials containing Ru nanoparticles with bare ones did not show any measurable difference, indicating that Ru nanoparticles do not contribute to heating.

The size distribution of the Ru nanoparticles was narrow, with an average diameter of d = 1.6 ± 0.4 nm (Figure 2c,d). Free Ru nanoparticles (not attached to the support) were not observed. In addition to nanoparticles, clusters of Ru atoms were also formed (marked in Figure 2d with arrows). The measured amount of elemental Ru (5.3 wt.%) in AC-Ru is identical to the amount calculated from the mass balance, which indicates a complete reduction of Ru^3+^. In the magnetically mediated synthesis of the AC-Ru, the temperature of the liquid never exceeded 100 °C (Table S1). Our unpublished study showed that the reduction of Ru(C_5_H_7_O_2_)**_3_** in 2-propanol proceeds only above 130 °C. The above suggests that the surface of the MN-C had rapidly been heated to a temperature above 130 °C and that the relatively thin film of liquid surrounding the heated particles remained at a high temperature, so Ru(C_5_H_7_O_2_)**_3_** was completely reduced to Ru^0^ and nucleated exclusively on the surface as depicted in Figure 2e. For comparison, the nanocatalyst CH-Ru was synthesized using conventional heating (see Supplementary Materials Section S1). The pressure vessel and its content were heated to 150 °C using an oil bath (see Supplementary Materials Section S1). The solution became colorless after 30 min, indicating a slower reduction kinetics as in the case of the magnetically mediated synthesis of AC-Ru. The slower kinetics further suggests that the surface must have been heated above 150 °C during the synthesis of AC-Ru. Ru nanoparticles with a diameter of ~3 nm were homogeneously distributed over the surface of CH-Ru (Figure 2f,g). In addition to the individual nanoparticles, agglomerates of the Ru nanoparticles were also formed, which indicate homogeneous nucleation of the Ru nanoparticles in the bulk of the heated 2-propanol (Figure 2f,g and Figure S6). The weight fraction of the deposited Ru in the CH-Ru was ~3 wt.%, lower than expected from the mass balance, suggesting that a significant fraction of the Ru nanoparticles was removed during washing of the CH-Ru after synthesis.

**Figure 3:**
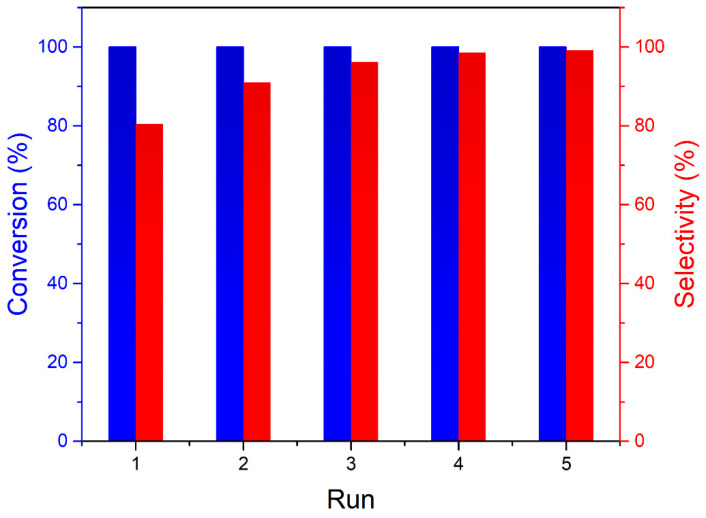
Conversion of furfural and selectivity for the furfuryl alcohol over the AC-Ru nanocatalyst in five consecutive runs. (More details in Supplementary Materials Section S1) Reaction conditions: AC-Ru (48 mg), furfural (0.1 g, 1.04 mmol), 2-propanol (0.8 mL), H_2_, 1 MPa, 90 °C, 3 h.

The catalytic test in a pressurized stirred slurry reactor showed a very high activity of AC-Ru nanocatalyst for furfural hydrotreatment in 2-propanol. Furfural that is produced from xylose is regarded as a versatile and renewable chemical with a wide range of industrial applications [27,28,29,30]. Conversion to the furfuryl alcohol compound used mainly in the polymer industry is a simple catalyzed hydrogenation reaction that consumes nearly 60% of furfural produced worldwide [27,28,29,30]. In the first run, the conversion of furfural over AC-Ru was 100% in 3 h, even at a very low reaction temperature of 90 °C, while selectivity for furfuryl alcohol was 80% (Figure 3). The side products were tetrahydrofurfuryl alcohol (18%) and methyl furan (2%), indicating very high activity of the AC-Ru. Recyclability of the AC-Ru was tested in another four consecutive runs. Conversion remained at 100%, but selectivity for furfuryl alcohol increased to 99.1% in the fifth run (Figure 3), demonstrating high activity of AC-Ru for aldehyde group hydrogenation, while it kept the five-membered heterocyclic ring intact. The high activity and selectivity of the AC-Ru strongly suggest that the metallic Ru nanoparticles were the catalytically active phase [27,28,29,30]. The increase of selectivity was most likely related to a slight decrease of activity in the most active sites responsible for over hydrogenation and dehydration. Catalytic activity of the CH-Ru was, despite lower fraction of Ru, remarkably good. In the first run, the conversion was 100% and selectivity for the furfuryl alcohol was 95% (Figure S7). Detailed spectrometric investigation (H^1^ NMR and QMS) of the products revealed that the remaining 5 mol% of the products was formed via the competitive reaction between furfural and isopropanol in the bulk liquid; specifically, acetalization reaction took place yielding furfural diisopropyl acetal (CAS 187995-47-7). Its formation was detected in blank test runs in absence of the catalyst; therefore, its formation in the bulk liquid could only be further reduced with an increase of the catalyst loading or completely omitted using a suitable solvent without the hydroxyl functional group. The catalyst CH-Ru remained active in the second run with similar selectivity and product distribution (Figure S7).

## 4. Conclusions

In conclusion, we have shown a magnetically mediated process for the rapid synthesis of a Ru-based nanocatalyst. The locally delivered heat led to homogeneous deposition of Ru nanoparticles with a diameter of 1.6 nm exclusively on the surface of the support. Compared to conventional heating, the synthesis time for nanocatalyst preparation was significantly shortened; the magnetically mediated heating up period was practically immediate; the time to quantitatively deposit Ru was significantly decreased and the cooling down period was immediate, since the bulk of the liquid medium remained at a significantly lower temperature. The process, although far from being optimized, offers utilization of electricity for the rapid synthesis of this functional material. The synthesized nanocatalyst showed remarkable activity in the hydrogenation of furfural to furfuryl alcohol. After four recycling cycles, the conversion remained at 100%, while selectivity was steadily increased from 80% in the first run to >99% in the last.

## Supplementary Materials

The following are available online at https://www.mdpi.com/2079-4991/10/6/1142/s1, Table S1: Vessel’s surface temperature, pressure and solution color as the function of time into AC-field heating, Figure S1: Vector representation of the *μ*_0_*H* on a plane through the center of the inductor coil, Figure S2: Color map of the *μ*_0_*H* magnitude on the plane through the center of the coil, Figure S3: XRD powder pattern of the MN-C, Table S2: Estimated crystallite size and weight fraction of the corresponding crystalline phase, room-temperature magnetic properties and specific surface area of the MN-C, Figure S4: BF and HAADF STEM images of the MN-C, Figure S5: Raman spectrum of the MN-C, Figure S6: BF and HAADF STEM images of the CH-Ru, Figure S7: Recycling of the CH-Ru nanocatalyst.
