# Supplementary material for: Magnetic Heating of Nanoparticles Applied in the Synthesis of a Magnetically Recyclable Hydrogenation Nanocatalyst
Source: Nanomaterials (Basel). 2020 Jun 10;10(6):1142. doi: 10.3390/nano10061142 (PMC7353275; doi:10.3390/nano10061142)
Supplement: Supplementary file 1 [file nanomaterials-10-01142-s001.pdf]

nanomaterials

Supplementary Materials

Magnetic Heating of Nanoparticles Applied in the Synthesis of a Magnetically Recyclable Hydrogenation Nanocatalyst

Sašo Gyergyek [1,*], Darja Lisjak [1], Miloš Beković [2], Miha Grilc [3], Blaž Likozar [3], Marijan Nečemer [4] and Darko Makovec [1]

1 Department for Materials Synthesis, Jožef Stefan Institute, Jamova 60, 1000 Ljubljana, Slovenia; darja.lisjak@ijs.si (D.L); darko.makovec@ijs.si (D.M.)
2 Institute of Electrical Power Engineering, Faculty of Electrical Engineering and Computer Science, University of Maribor, Koroška 46, 2000 Maribor, Slovenia; milos.bekovic@um.si
3 Department of Catalysis and Chemical Engineering, National Institute of Chemistry, Hajdrihova 19, 1000 Ljubljana, Slovenia; miha.grilc@ki.si (M.G), blaz.likozar@ki.si (B.L.)
4 Department for Low and Medium Energy Physics, Jožef Stefan Institute, Jamova 60, 1000 Ljubljana, Slovenia; marijan.necemer@ijs.si
* Correspondence: saso.gyergyek@ijs.si

S1. Experimental Details

S1.1. Magnetic Field Applicator

An Ambrell (Rochester, NY, USA) HTG-6000 (6 kW) high frequency generator was used as an AC-current source. Copper-tube coil (Induktio d.o.o., Ljubljana, Slovenia) with an inner diameter of 34 mm, a height of 43 mm and 5 turns resonated at 273 kHz when connected to the generator. Generator and coil were water cooled using a closed compressor cooler OBE 003 (Omega Air, Burlington, ON, Canada, 2.74 kW). The finite element method was used to calculate and visualize magnetic field distribution within the coil.

S1.2. Catalyst Support Synthesis (MN-C)

Citric-acid-coated magnetic iron oxide nanoparticles (MN) were synthesized using a previously published method with simple co-precipitation followed by the adsorption of the citrate ion in an aqueous solution [1]. The MNs were dispersed in deionized water, to which glucose was added. The final concentration was 9 g/L of MNs and 90 g/L of glucose. A concentrated solution of NaOH was used to adjust the pH to a value of approximately 10. A total of 800 mL of the suspension was transferred to a 1-litre stainless-steel Parr autoclave equipped with a stirrer. The suspension was treated at 180 °C for 12 h. The brown flock (MN-aC) was magnetically separated, washed several times with DDI water and dried at 80 °C in an oven. The dried powder was annealed in a tubular furnace at 600 °C for 6 h in an atmosphere of Ar (MN-C). The black powder was grinded as a 2-propanol slurry in an agate mortar, dried under vacuum and stored in a desiccator.

S1.3. Catalyst Synthesis

S1.3.1. Magnetically Mediated Synthesis of the Catalyst AC-Ru

Sixty milligrams of the MN-C was transferred to the round bottom glass pressure vessel (Q-Tube-Purging -35-SS, LabTech™, East Lime, CT, USA and 3 mL of 0.01 M Ru (III) 2,4-pentadionate was added. The vessel was purged with Ar for 30 min and finally pressurized with Ar to 10 bar. The vessel was placed in the centre of the inductor coil and the field of $\mu_0H = 86$ mT was turned on. After a couple of minutes of AC heating the field was turned off, the vessel was removed from the coil, the

surface temperature of the vessel was measured using FLIR™ IR camera (Wilsonville, OR, USA), the MN-C was magnetically sedimented and the color of the solution was checked.

Table S1. Vessel's surface temperature, pressure and solution colour as the function of time into the AC-field heating.

t/min	*T*/°C	*p*/bar	Colour
0	RT	10	pink
7	74	11*	lighter pink
14	99	11*	slightly pink
19	77	10	colourless

* Pressure vessel safely withstands pressure up to 12.4 bar. Pressure was manually released to 10 bar when reached 11 bar during heating.

After 19 min, the vessel was removed from the coil and in approximately 3 min cooled to room temperature when it was opened and the AC-Ru was separated using a handheld permanent magnet. The AC-Ru was washed 5 times with pure 2-propanol and dried in a vacuum at room temperature. Based on XRF analysis, the AC-Ru contained 5.3 ± 0.6 wt.% of Ru.

S1.3.2. Synthesis of the Catalyst CH-Ru (Conventional Heating)

Sixty milligrams of the MN-C was transferred to the round bottom glass pressure vessel (Q-Tube-Purging -35-SS, LabTech™) and 3 mL of 0.01 M Ru (III) 2,4-pentadionate was added. The vessel was purged with Ar for 30 min and finally pressurized with Ar to 10 bar. The vessel was placed in an oil bath and heated to 150 °C. Every 10 min the vessel was removed from the oil bath, the MN-C was magnetically sedimented and the colour of the solution was checked. After 30 min the solution became colourless. The CH-Ru was washed 5 times with pure 2-propanol and dried in vacuum at room temperature. Based on X-ray fluorescence spectrometer (XRF) analysis, the CH-Ru contained 2.6 ± 0.3 wt.% of Ru.

S1.4. Hydrogenation of Furfural

A total of 48 mg of the catalyst was weighted into the round bottom glass pressure vessel (Q-Tube-Purging -35-SS, LabTech™), and 0.1 g of furfural and 0.8 g of isopropanol were added. The vessel was closed, purged with H_2 gas and pressurized with H_2 to 10 bar. The vessel was placed in an oil bath and heated for 3 h at 90 °C during vigorous stirring (800 rpm). After 3 hours, the vessel was left to cool naturally to room temperature. The catalyst was separated using a handheld permanent magnet and the clear liquid part was decanted. The liquid was analyzed by GC-QMS (Ultra 2010, Shimadzu, Japan) after dilution with acetone (>99.9% Sigma-Aldrich). When the CH-Ru was used as a catalyst, additional characterization was done using the benchtop ^{1}H NMR (Spinsolve 60, Magritek, Wellington, New Zealand). The catalyst was washed 5 times with pure isopropanol and dried in a vacuum at room temperature. The AC-Ru catalyst was recycled 4 times (5 hydrogenations in total using the same catalyst) and the CH-Ru 1 time (2 hydrogenations in total using the same catalyst).

S2. Results and Discussion

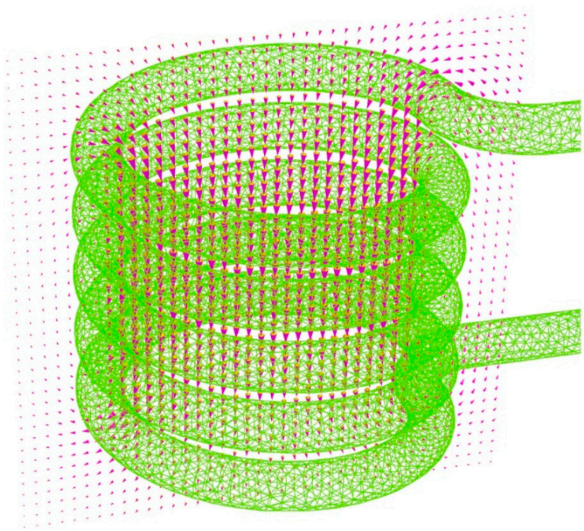

Figure S1. Vector representation of the $\mu_0 H$ on a plane through the center of the inductor coil.

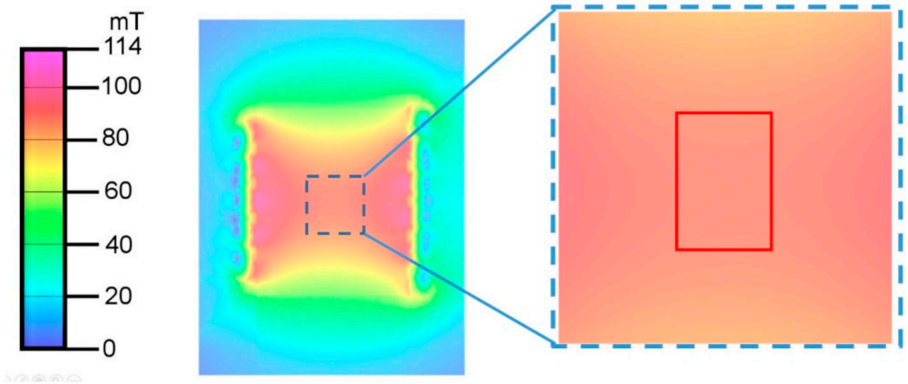

Figure S2. Color map of the $\mu_0 H$ magnitude on the plane through the center of the coil. The zoomed-in area is roughly larger than the size occupied by the sample during AC-Ru synthesis (in blue), and the red square represents the cross-section occupied by the samples for the SAR measurements.

Because the coil has finite dimensions, the magnetic field inside the coil is not perfectly homogeneous (Figures S1 and S2). The field distribution inside the coil was calculated by a finite element method using Opera software (Figures S1 and S2). The field within the region of the coil that samples were exposed to varies for ~7% with respect to the field in the center of the coil (Figure S2). The AC-magnetic field amplitude $\mu_0 H$ is the integral over the surface centered in the coil and has the same dimensions as the sample's cross-section divided by the same surface. In this way, the $\mu_0 H$ is understood as the average field amplitude that the sample volume is exposed to.

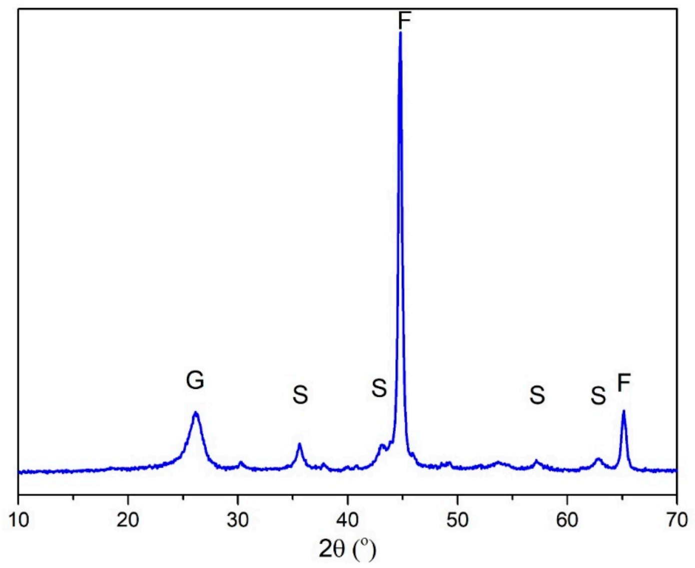

Figure S3. XRD powder pattern of the MN-C. S corresponds to reflections of cubic spinel (iron oxide) (space group Fd-3m), F of BCC iron (space group Im-3m) and G of graphite (space group P63mc). Very low-intensity, barely visible reflections belong to orthorhombic cohenite Fe_3C (space group Pnma) and cubic iron oxide wuestite (space group Fm-3m).

Table S2. Estimated crystallite size and weight fraction of the corresponding crystalline phase, room-temperature magnetic properties and specific surface area of the MN-C.

Sample	d_{XRD}(nm)/wt.%			Magnetic properties			Surface area	
	S	F	G	M_s(emu/g)	M_r (emu/g)	H_c (Oe)	S_{BET} (m^2/g)	V_p(cm^3/g)
**MN-C**	14/10	54/26	5/64	31.9	8.1	410	258	0.379

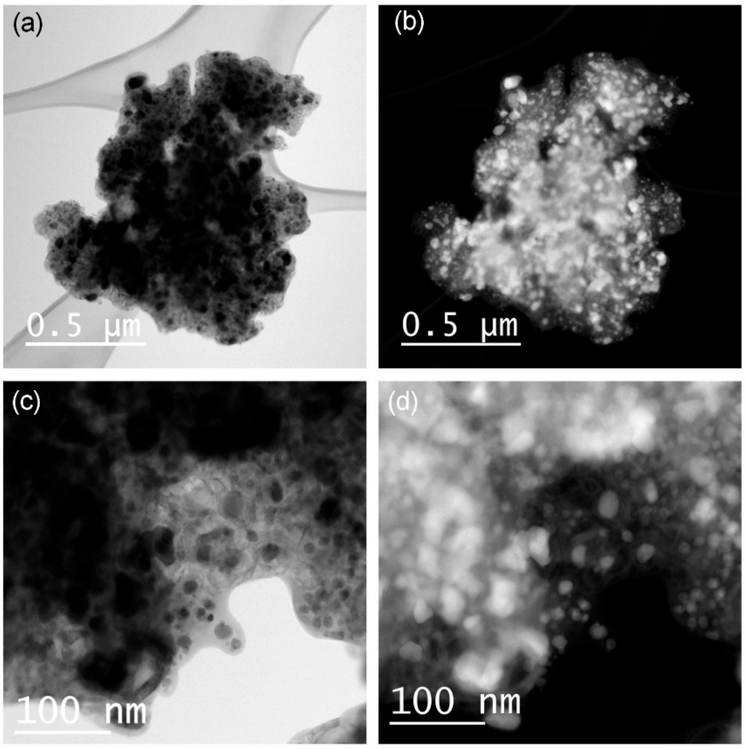

Figure S4. BF STEM image of the MN-C at **(a)** lower and **(d)** higher magnification; HAADF STEM image of the MN-C at **(b)** lower and **(d)** higher magnification.

S2.1. Raman spectroscopy

The first-order Raman spectrum of the MN-C shows two broad peaks that are clearly composed of 5 individual bands (Figure S5). The molecular approach to interpretation of Raman spectra associates G band to stretching of all pairs of sp^2 C atoms in rings and chains [2], D1 to breathing modes of sp^2 C atoms in rings [2], D2 to edges of graphitic crystallites [3], D3 to amorphous C phase [4,5] and D4 to vibrations of polyenes [6]. Usually the ratio of I(D)/I(G) is used to estimate crystal size of graphite; however, in the presence of sp^3 C phases and when the expected size is significantly below 100 nm, the excitation-energy-independent G bands' half-width-at-half-maximum (HWHM) is used to estimate the lateral size of the graphite domain L_a, what is usually interpreted as the crystallite size of nanographite [2,7]. In the sample MN-C600-6, the L_a was found to be 7.5 nm.

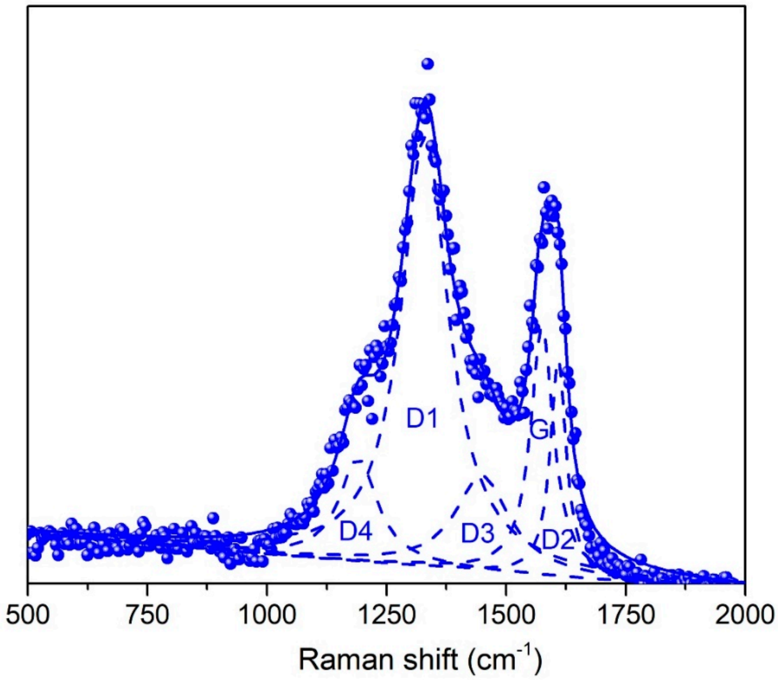

Figure S5. Raman spectrum of the MN-C. Dots represent experimental data, dashed lines individual bands fitted with Lorentzian function, and a full line is a sum of Lorentzian functions.

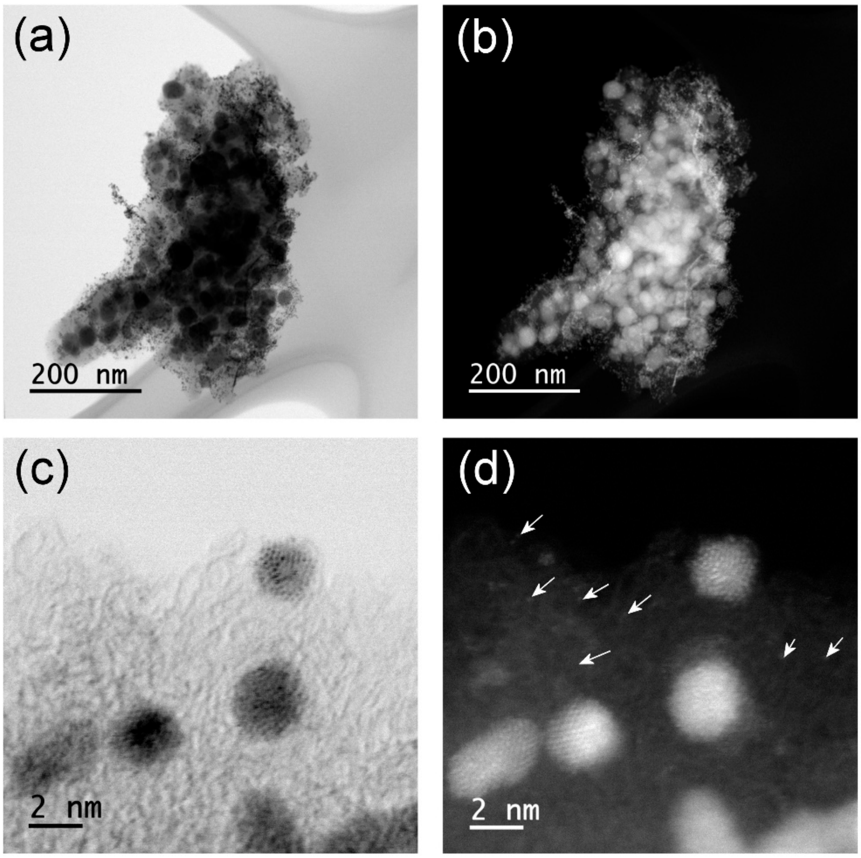

Figure S6. BF STEM image of the CH-Ru at (**a**) lower and (**d**) higher magnification. HAADF STEM image of the CH-Ru at (**b**) lower and (**d**) higher magnification. (**d**) White arrows mark Ru atom/clusters.

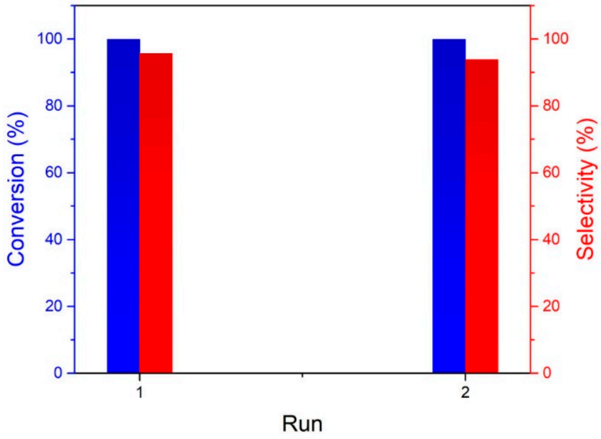

Figure S7. Conversion of furfural and selectivity for the furfuryl alcohol over the CH-Ru nanocatalyst in two consecutive runs. Reaction conditions: CH-Ru (48 mg), furfural (0.1 g, 1.04 mmol), 2-propanol (0.8 mL), H_2, 1 MPa, 90 °C, 3 h.

References

1. Nečemer, M.; Kump, P.; Vogel-Mikuš, K. *Handbook of Phytoremediation*; Nova Science Publishers: New York, USA, 2011.
2. Ferrari, A.C.; Robertson, J. Raman spectroscopy of amorphous, nanostructured, diamond-like carbon, and nanodiamond. *Phil. Trans. R. Soc. Lond. A* **2004**, *362*, 2477.
3. Gançado, L.G.; Pimenta, M.A.; Neves, B.R.; Dantas, M.S.; Joira, A. Influence of the atomic structure on the raman spectra of graphite edges. *Phys. Rev. Lett.* **2004**, *93*, 247401.

4. Cuesta, A.; Dhamelincourt, P.; Laureyns, J.; Martinez-Alonso, A.; Tascon, J.M.D. Raman microprobe studies on carbon materials. *Carbon* **1994**, *32*,1523.
5. Jawhari, T.; Roid, A.; Casado, J. Raman spectroscopic characterization of some commercially available carbon black materials. *Carbon* **1995**, *33*,1561.
6. Dipper, B.; Jander, H.; Heintzenberg, J. NIR FT Raman spectroscopic study of flame soot. *Phys. Chem. Chem. Phys.* **1999**, *1*, 4707.
7. Mallet-Ladeira, P.; Puech, P.; Toulouse, C.; Cazayous, M.; Ratel-Ramond, N.; Weisbecker, P.; Vignoles, G.L.; Monthioux, M. A Raman study to obtain crystallite size of carbon materials: A better alternative to the Tuinstra-Koenig law. *Carbon* 2014, **80**, 629.
